# A subpopulation of astrocyte progenitors defined by Sonic hedgehog signaling

**DOI:** 10.1186/s13064-021-00158-w

**Published:** 2022-01-14

**Authors:** Ellen C. Gingrich, Kendra Case, A. Denise R. Garcia

**Affiliations:** 1 Present Address: Department of Biology, Stanford University, Stanford, CA 94305 USA; 2 Drexel University, 3245 Chestnut St. PISB 422, Philadelphia, PA 19104 USA; 3 Present Address: Department of Neurobiology and Anatomy, Drexel University College of Medicine, Philadelphia, PA 19129 USA

**Keywords:** Astrocyte, Sonic hedgehog, Gli1, Glia, Cortex, Fate mapping

## Abstract

**Background:**

The molecular signaling pathway, Sonic hedgehog (Shh), is critical for the proper development of the central nervous system. The requirement for Shh signaling in neuronal and oligodendrocyte development in the developing embryo are well established. However, Shh activity is found in discrete subpopulations of astrocytes in the postnatal and adult brain. Whether Shh signaling plays a role in astrocyte development is not well understood.

**Methods:**

Here, we use a genetic inducible fate mapping approach to mark and follow a population of glial progenitor cells expressing the Shh target gene, *Gli1*, in the neonatal and postnatal brain.

**Results:**

In the neonatal brain, *Gli1*-expressing cells are found in the dorsolateral corner of the subventricular zone (SVZ), a germinal zone harboring astrocyte progenitor cells. Our data show that these cells give rise to half of the cortical astrocyte population, demonstrating their substantial contribution to the cellular composition of the cortex. Further, these data suggest that the cortex harbors astrocytes from different lineages. Gli1 lineage astrocytes are distributed across all cortical layers, positioning them for broad influence over cortical circuits. Finally, we show that Shh activity recurs in mature astrocytes in a lineage-independent manner, suggesting cell-type dependent roles of the pathway in driving astrocyte development and function.

**Conclusion:**

These data identify a novel role for Shh signaling in cortical astrocyte development and support a growing body of evidence pointing to astrocyte heterogeneity.

**Supplementary Information:**

The online version contains supplementary material available at 10.1186/s13064-021-00158-w.

## Introduction

Astrocytes encompass a diverse population of cells that possess a broad array of functional properties that are essential for nervous system function. They are responsible for neurotransmitter clearance, ion buffering, synapse formation, maintenance of the blood brain barrier, and provide energetic support for neurons [1]. The developmental processes that confer their functional characteristics are not well understood. Astrocyte production and maturation occurs primarily during the initial weeks after birth, coinciding with synapse formation and the refinement of neural circuits. Indeed, growing evidence supports a role for astrocytes in the establishment and plasticity of developing neural circuits [2–5]. While a large body of work has delineated molecular mechanisms underlying astrocyte specification during embryonic development, the processes surrounding postnatal astrocyte development, the time during which most astrocytes are generated, are considerably less well defined.

The Sonic hedgehog (Shh) signaling pathway is best characterized during embryonic neurodevelopment, where it exerts powerful influence over a broad array of neurodevelopmental processes, including patterning and morphogenesis, axon pathfinding, and cell type specification of ventral motor neurons and oligodendrocytes [6]. Despite the well-established roles for Shh signaling in neuron and oligodendrocyte development, considerably less is known about its role in astrocyte development. In the developing optic nerve, SHH derived from retinal ganglion cells promotes proliferation of astrocyte precursors [7]. In contrast, SHH in the early embryonic spinal cord limits the specification of astrocyte progenitor cells [8]. In the adult brain, Shh signaling is found predominantly in astrocytes [9], where it regulates expression of genes such as channels and receptors that mediate astrocyte modulation of synapses [3, 10]. Whether Shh signaling plays a role in astrocyte development is not well understood.

Astrocyte production occurs primarily during the first 2 weeks of postnatal development [11]. They are derived from radial glia and progenitor cells in the ventricular zone (VZ) and subventricular zone (SVZ) [12–16], respectively, as well as from local proliferation of differentiated astrocytes [17]. The dorsal region of the SVZ harbors progenitor cells that generate both oligodendrocytes and astrocytes [13, 18]. The postnatal ventricular and subventricular zones (V-SVZ) harbors a population of progenitor cells that express *Gli1*, a transcriptional target of Shh signaling [19, 20]. Gli1-expressing progenitors generate a substantial population of oligodendrocytes that populate the overlying white matter [18]. Whether Gli1 progenitors in the V-SVZ are also responsible for generating astrocytes is not known.

In this study, we performed fate mapping of progenitor cells expressing *Gli1* in the postnatal brain to determine whether these cells generate cortical astrocytes and mapped their distribution across cortical layers. We found that a subpopulation of astrocyte progenitor cells in the SVZ express *Gli1*. Astrocytes within the Gli1 lineage comprise half of the total cortical astrocyte population, demonstrating a substantial contribution of *Gli1*-expressing astrocyte progenitor cells to the cellular assembly of the cortex. These data further suggest that the cortex harbors multiple populations of astrocytes from different lineages, suggesting that this may be one mechanism by which astrocytes achieve functional diversity.

## Results

### Astrocytes in the Gli1 lineage are broadly distributed in the cortex

**Fig. 1:**
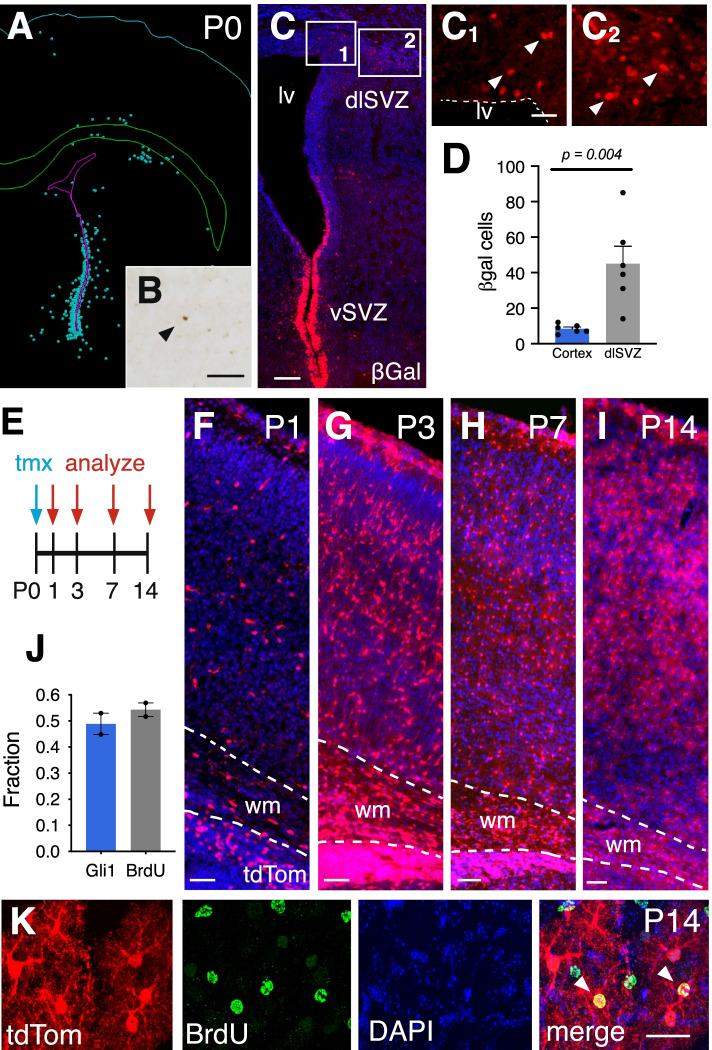
The population of Gli1 cells expands during postnatal development. **A-D** The distribution of βGal-labeled cells in *Gli1*^*nlacZ/+*^ tissues at P0. (A) Neurolucida tracing from brightfield immunostained sections. Each dot represents a single cell. **B-C** Brightfield (B) and immunofluroescent (C) staining of βGal at P0 in the cortex (B) and SVZ (C). Insets in C shown in C_1_ and C_2_. Scale bars, B, C_1_-C_2,_ 50 μm, C, 100 μm. lv, lateral ventricle, vSVZ, ventral SVZ, dlSVZ, dorsolateral SVZ. **D** The number of βGal-labeled cells in the cortex and dlSVZ at P0. Bars represent mean ± SEM, data points represent individual animals (*n = 6*). Statistical significance assessed by unpaired Student’s t-test. **E** Schematic depicting experimental timeline. **F-I** TdTom (red) expression in the cortex of *Gli1*^*CreER/+*^;Ai14 mice that received tamoxifen at P0 and analyzed at P1 (F), P3 (G), P7 (H), and P14 (I) shows a broad distribution of marked cells throughout the cortex that expands over time. Counterstaining with DAPI (blue), wm, white matter. Scale bar, 50 μm. **J** The fraction of marked cells (Gli1) and dividing cells (BrdU) that are double labeled in mice marked at P0 and analyzd at P14 (*n = 919* Gli1 and *n = 820* BrdU cells, from 2 animals). Bars represent mean ± SEM, data points represent individual animals. **K** Immunofluorescent staining for BrdU in the cortex of a mouse at P14 that received tamoxifen at P0. Single channel and merged max projection images from confocal stacks in the cortex showing colocalization of marked cells (red) and BrdU (green). Counterstaining with DAPI (blue). Arrowheads, double labeled cells. Scale bar, 25 μm

We first examined the expression of *Gli1* in the early postnatal mouse cortex in *Gli1*^*nlacZ/+*^ mice carrying a nuclear lacZ in the *Gli1* locus [21]. At postnatal day 0 (P0), there was a pronounced population of *Gli1*-expressing cells in the ventral SVZ, corresponding to the population of SVZ precursors that generate deep granule neurons and periglomerular cells in the olfactory bulb [20, 22, 23] (Fig. 1). *Gli1*-expressing cells were also observed in the dorsolateral corner of the SVZ (dlSVZ), within the postnatal germinal zone of cortical astrocytes [13], and in a region inferior to the white matter overlying the ventricles (Fig. 1). Few cells were observed in the cortex at this age (Fig. 1). We quantified and mapped the distribution of βGal-labeled cells within the dlSVZ and cortex and found that the cortex harbored relatively few *Gli1*-expressing cells at P0 compared to the dlSVZ (Fig. 1).

*Gli1*-expressing progenitors residing in the subcallosal dorsolateral domain of the SVZ (dlSVZ) generate a large fraction of oligodendrocytes that populate the corpus callosum and overlying white matter tract [18]. Retroviral labeling experiments demonstrate that this region also generates glial cells that populate the cortex [12]. To determine whether *Gli1*-expressing progenitors also contribute glial cells to the cortex, we performed fate mapping in *Gli1*^*CreER/+*^ mice [19] carrying the Ai14 tdTomato (tdTom) reporter [24] (*Gli1*^*CreER/+*^;Ai14). Cre-mediated recombination promotes expression of tdTom that is both permanent and heritable. We marked *Gli1*-expressing precursors by administering tamoxifen to *Gli1*^*CreER/+*^;Ai14 mice at P0 and analyzed the distribution of tdTom at various postnatal ages. One day after tamoxifen (P1), there was a substantial population of marked cells in the cortex that were distributed across all layers (Fig. 1). At P3, there was a dramatic expansion of marked cells (Fig. 1), suggesting extensive proliferation between P1 and P3. We also observed many residual radial glial fibers and cells with transitional morphologies, consistent with radial glia undergoing transformation into multipolar astrocytes (Supplemental Fig. 1). These cells co-express vimentin, a marker of radial glia (Supplemental Fig. 1), suggesting that in addition to progenitors residing in the dlSVZ, Shh also signals to radial glial progenitors. There was a further expansion observed at P7 (Fig. 1), suggesting that *Gli1*-expressing cells marked at P0 correspond to actively dividing glial progenitor cells. There was no further expansion in the number of marked cells between P7 and P14, suggesting *Gli1*-expressing cells marked at birth proliferate largely during the first postnatal week. To confirm the observed expansion was due to proliferation, we administered BrdU to mice approximately 12 h after tamoxifen, to ensure sufficient Cre-mediated recombination prior to incorporation of BrdU. We analyzed tissues at P14 and found that 49% of marked cells in the cortex are co-labeled with BrdU (Fig. 1). Conversely, 54% of BrdU labeled cells co-expressed tdTom, indicating that only a fraction of proliferating glial precursors at P0 express *Gli1* (Fig. 1). These data show that *Gli1*-expressing cells residing within the dlSVZ generate cells that migrate into the cortex and expand locally.

### Gli1 progenitors generate a subpopulation of cortical astrocytes

**Fig. 2:**
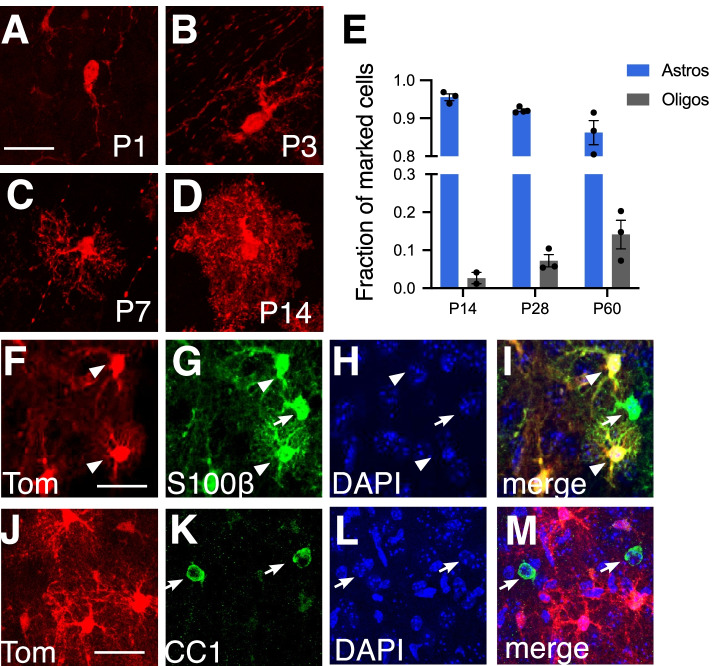
Cells expressing Gli1 at P0 generate cortical astrocytes. **A-D** Cells marked at P0 and analyzed at P1 (A), P3 (B), P7 (C), and P14 (D) show an increasingly complex morphology consistent with an astrocyte identity as development proceeds. **E** Single cell quantification of the fraction of marked cells that correspond to astrocytes or oligodendrocytes. Data points represent individual animals (*n = 2–4* animals). At least 150 cells/animal analyzed. **F-M** Single channel (F-H, J-L) and merged images (I, M) from confocal images of tdTom (F, J, red) and S100β (G, green) or CC1 (K, green) in the cortex at P14, following tamoxifen administration at P0. Marked cells show colocalization with S100β, but not with CC1. Counterstaining with DAPI (H, L, blue). Arrowheads, colocalized cells; arrows, single labeled astrocytes or oligodendrocytes not marked with tdTom. Scale bar, 25 um

We examined the morphologies of marked cells in the cortex and found that at early time points, marked cells showed simple morphologies, with one or two processes, consistent with an immature phenotype (Fig. 2). Over time, marked cells developed an increasingly complex morphology, extending multiple processes. By P14, cells exhibited several major primary branches together with many fine branchlets, conferring the typical bushy morphology of protoplasmic astrocytes (Fig. 2). We performed colocalization analysis with glial cell-type specific markers at P14. A small fraction of marked cells co-expressed the oligodendrocyte-specific marker, CC1 (Fig. 2), whereas the overwhelming majority were co-labeled with S100β, identifying them as astrocytes (Fig. 2). We also observed substantial expression of tdTom in the white matter overlying the ventricles in tissues marked as late as P7 (Figs. 1 and 4), consistent with *Gli1*-derived oligodendrocytes [18]. These data demonstrate that neonatal progenitors in the dlSVZ correspond to astrocyte progenitor cells responsible for contributing cortical astrocytes. Although the fraction of marked cells corresponding to oligodendrocytes at P14 was small, this fraction increased at P28 and P60 (Fig. 2), consistent with oligodendrocyte production during late postnatal development [11]. However the fraction of marked cells differentiating into oligodendrocytes remained small and never exceeded 14% (Fig. 2). Notably, marked oligodendrocytes in the cortex were only observed in mice that received tamoxifen at P0. Tamoxifen given to mice beyond P0 generated marked cells that were identified predominantly as astrocytes (Table 1). These data suggest that *Gli1*-expressing progenitor cells residing in the early postnatal dlSVZ correspond predominantly to astrocyte progenitors.

**Table 1:** Gli1 cells marked at various ages correspond to astrocyte progenitors or mature astrocytes

Age	Astrocytes
P0	95% (*783*)
P3	99% (*843*)
P7	99% (*578*)
P14	97% (*348*)
P28	98% (*389*)
Adult	99% (*191*)

Single cell analysis of the identity of marked cells in *Gli1*^*CreER/+*^;Ai14 mice that received tamoxifen at the ages shown in the left column, and analyzed two weeks later. The fraction of tdTom cells identified as astrocytes by colocalization with S100β. The number of individual cells analyzed is in parentheses. *n = 3–4* mice for each age

**Fig. 3:**
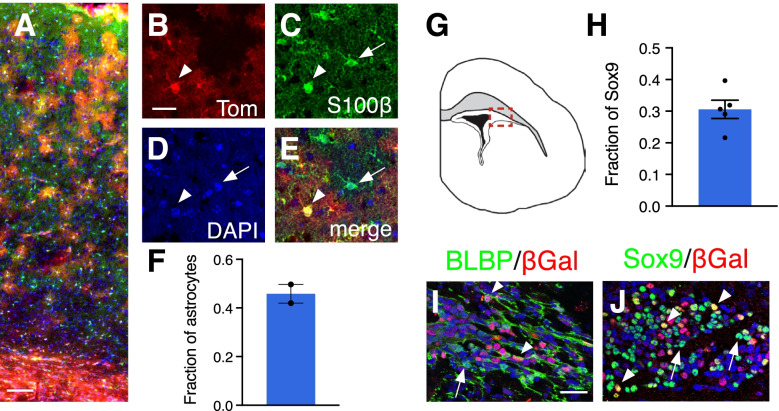
A subpopulation of astrocyte progenitors in the SVZ express Gli1. **A** Distribution of tdTom (red) in the cortex of adult (P60) *Gli1*^*CreER/+*^;Ai14 mouse marked at P0. Scale bar, 100 μm. **B-E** Single channel, confocal images of tdTom (B, red) and S100β (C, green) identifying marked cells as astrocytes. Counterstained with DAPI (D, blue). Merged image shown in E. Arrowhead, double labeled cell; arrows, single labeled cells. Scale bar, 25 μm **F** The fraction of cortical astrocytes in the Gli1 lineage (*n = 1371* cells from 2 animals). **G** Tracing of a P0 brain section highlighting the dlSVZ (red inset). **H** The fraction of Sox9 labeled cells in the dlSVZ co-labeled with βGal at P0 (*n = 8358* cells from 5 animals). **I-J** Double labeling for βGal (red) and the astrocyte progenitor markers BLBP (I, green) or Sox9 (J, green) in the dlSVZ of *Gli1*^*nlacZ/+*^ mice at P0. Counterstained with DAPI (blue). Scale bar, 25 um. Images taken from red inset shown in G. Bars represent mean ± SEM, data points represent individual animals

We next investigated the extent to which astrocytes in the Gli1 lineage contribute to the total astrocyte population in the mature cortex. We administered tamoxifen at P0 and analyzed the fraction of cells labeled with the pan-astrocytic marker, S100β, that co-express tdTom at P60. We found that 46% of astrocytes were co-labeled with tdTom (Fig. 3), indicating that astrocytes within the Gli1 lineage make up nearly half of the total cortical astrocyte population. This suggests that the cortex harbors a mixed population of astrocytes from different lineages. These data further suggest that *Gli1*-expressing astrocyte progenitor cells comprise a subpopulation of the total pool of astrocyte progenitors. Alternatively, these data could reflect limitations of tamoxifen-dependent Cre-mediated recombination. To rule out the possibility that recombination was inefficient within the *Gli1*-expressing progenitor population, we examined the dlSVZ of *Gli1*^*nlacZ/+*^ mice, enabling us to identify cells actively expressing *Gli1* independent of the requirement for recombination. We identified the pool of astrocyte progenitors at P0 by Sox9 expression and found that 31% co-expressed *Gli1* (Fig. 3), consistent with the idea that progenitor cells expressing *Gli1* comprise a subpopulation of astrocyte progenitors in the P0 dlSVZ. This was confirmed with a second astrocyte progenitor marker, BLBP, in which we found that many BLBP labeled cells were *Gli1* negative (Fig. 3). Taken together, these data suggest that the astrocyte progenitor pool in the early postnatal dlSVZ is comprised of a molecularly distinct subpopulation that can be defined by Shh signaling.

### Shh signaling recurs in a subpopulation of differentiated astrocytes

**Fig. 4:**
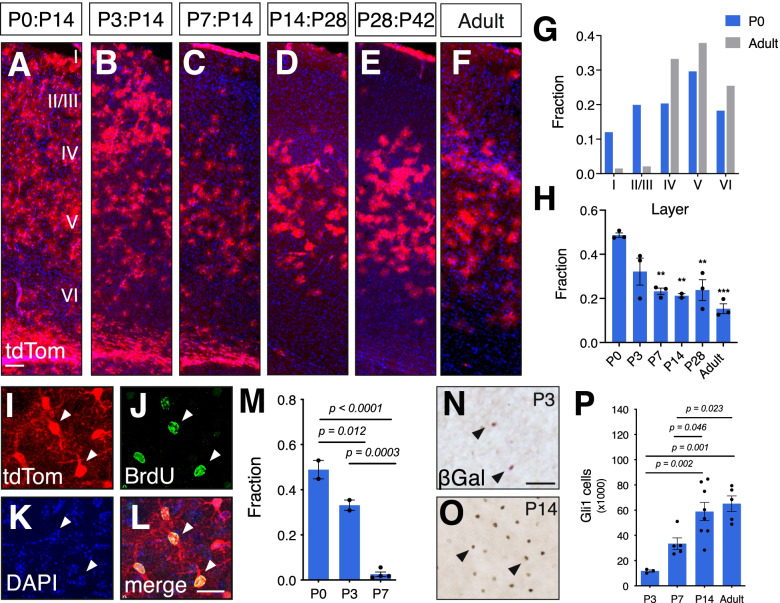
Gli1 cell distribution becomes restricted over postnatal development. **A-E** tdTom (red) expression in *Gli1*^*CreER/+*^;Ai14 tissues that received tamoxifen at P0 (A), P3 (B), P7 (C), P14 (D), P28 (E) and as adults (F) and analyzed at ages shown. Adult mice were analyzed two weeks after tamoxifen. Counterstaining with DAPI (blue). Scale bar, 100 μm. **G** The fraction of marked cells in each layer in mice that received tamoxifen at P0 or in adulthood. **H** The fraction of all astrocytes across all layers that are marked at various ages. Bars represent mean ± SEM, data points represent individual animals (*n = 3* animals per age). Statistical analysis performed by one-way ANOVA with Tukey’s post-hoc test for multiple comparisons. ** *p* < 0.005, ** *p* < 0.0005 compared to P0. **I-L** Single channel, confocal images of tdTom (I, red) and BrdU (J, green) identifying marked cells that are proliferating. Counterstained with DAPI (K, blue). Merged image shown in L. Scale bar, 25 μm. **M** The fraction of marked cells colabeled with BrdU at P14 in tissues marked at P0, P3 and P7 (*n = 919* cells, *n = 720* cells, *n = 566* cells, respectively, from 2 to 4 animals). Bars represent mean ± SEM, data points represent individual animals. Statistical analysis performed by one-way ANOVA with Tukey’s post-hoc test for multiple comparisons. **N-O** Brightfield immunostaining for βGal in *Gli1*^*nlacZ/+*^ tissues from mice at (N) P3 and (O) P14. Scale bar, 50 μm. **P** Estimated total number of Gli1-expressing cells derived from stereological quantification of βGal labeled cells in the cortex of *Gli1*^*nlacZ/+*^ mice at various ages. Bars represent mean ± SEM, data points represent individual animals (*n* = *3–8* animals per age). Statistical analysis performed by one-way ANOVA with Tukey’s post-hoc test for multiple comparisons

Our fate mapping studies showed that astrocytes within the Gli1 lineage are distributed ubiquitously throughout all layers of the neocortex. In contrast, Shh activity in the mature cortex is found predominantly in astrocytes localized to layers IV and V [3, 25]. To determine the age at which the distinctive laminar pattern of Shh signaling in the cortex emerges, we administered tamoxifen over three consecutive days at various ages during postnatal development. Tissues were analyzed 2 weeks after the initial tamoxifen dose, with the exception of P7 animals which were analyzed at one and 3 weeks after tamoxifen. There was no difference between these chase periods and the data were pooled for P7. In mice that received tamoxifen at P3, marked cells were observed throughout all cortical layers, showing a similar distribution as that seen at P0 (Fig. 4). However by P7, fewer marked cells are found in the upper cortical layers than in tissues marked at younger ages (Fig. 4). At P14, marked cells are found predominantly in deep layers and are largely absent from superficial layers, a pattern that was consistent with that observed at P28 and beyond (Fig. 4). Single cell analysis with cell-type specific markers showed that the vast majority of marked cells labeled at all ages were subsequently identified as astrocytes (Table 1). Quantitative analysis of the fraction of marked cells in each layer shows that tamoxifen labeling at P0 produces marked cells throughout all layers, with a moderate bias towards layer 5, which harbors 30% of marked cells, whereas layer 1 harbors only 12%. In contrast, the vast majority of marked cells are found in deep layers when tamoxifen is administered to adult mice (Fig. 4). This shift in marked cell distribution across development was associated with a concomitant reduction in the fraction of astrocytes, identified by S100β, that are marked at these ages (Fig. 4). At P0, 49% of astrocytes across all layers are marked, but by P3, that proportion declined to 32%, though this was not significant (Fig. 4). At P7, the fraction of marked astrocytes declined significantly from P0 to 23%. This fraction remained steady at P14 and P28 (21 and 24%, respectively, Fig. 4). There was a further modest reduction to 15% in adults, though this was not significant (Fig. 4). Double labeling with BrdU administered 12 h after tamoxifen shows that the fraction of marked cells that are dividing declines dramatically over the first postnatal week. At P0, the fraction of marked cells double labeled with BrdU was 49%, whereas at P3, that fraction was significantly reduced to 33%. By P7, nearly all marked cells were postmitotic as only 3% of tdTom cells co-labeled with BrdU (Fig. 4). This suggests that astrocytes within the Gli1 lineage are generated predominantly during the first few days after birth.

Despite the progressive decline in the fraction of astrocytes that are marked during the first postnatal week, analysis of active Shh signaling in the cortex in *Gli1*^*n*^^*lacZ/+*^ mice shows a progressive increase over the first two postnatal weeks (Fig. 4). Stereological quantification of the number of βGal labeled cells in the cortex showed a significant increase in the number of cells at P14 compared to P3 (Fig. 4). There was no difference between P14 and the adult (>P90) cortex (Fig. 4), suggesting that Shh signaling in the cortex stabilizes by the second postnatal week. Despite the increase in the number of cells exhibiting Shh activity between P7 and adulthood, few cells marked at P7 are proliferating (Fig. 4), arguing against the possibility that the increase in *Gli1*-expressing cells between P7 and adulthood is due to proliferation of immature progenitor cells. These data suggest that Shh signaling in astrocyte progenitor cells residing in the SVZ is transient and is lost as cells migrate into the cortex and undergo maturation. In parallel, Shh activity in the cortex is low at birth, but increases during postnatal development in a subpopulation of mature, postmitotic astrocytes found mostly in deep layers, coincident with the localization of Shh-expressing neurons in layer V [26, 27]. Taken together, these data suggest that Shh activity in neonatal astrocyte progenitor cells declines in immature astrocytes, but recurs in a subpopulation of postmitotic astrocytes localized primarily in layers IV and V.

### Recurrence of Shh signaling is independent of lineage

**Fig. 5:**
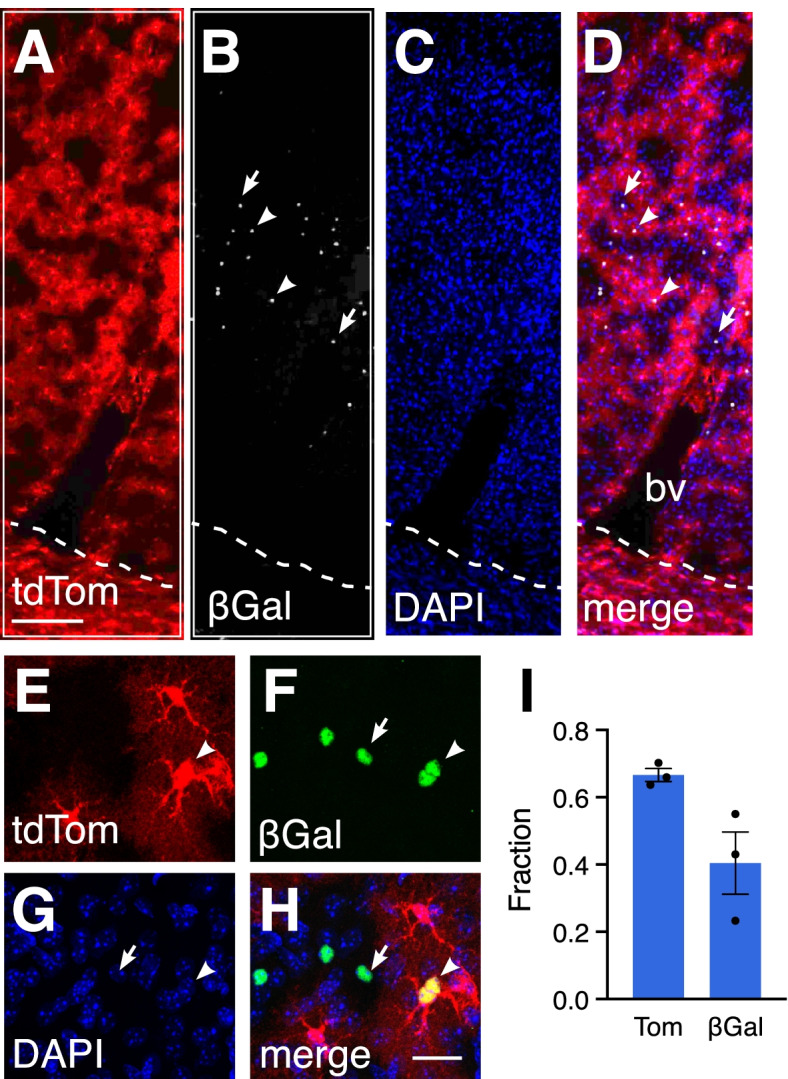
Shh signaling is upregulated in mature astrocytes (**A-H**) Double labeling for tdTom (A, E, red) and βGal (B, grey, F, green) in the cortex of a *Gli1*^*CreER/nLacZ*^;Ai14 mouse marked at P0 and analyzed at P60. (A-D) Low magnification epifluorescent and (E-H) high magnification confocal images. Counterstained with DAPI (C, G, blue). Dotted line depicts white matter border. Merged images shown in D, H. bv, blood vessel. Arrowheads, colocalized cells; arrows, βGal only cells. **I** The fraction of tdTom or βGal labeled cells that are single labeled (*n = 1454* and *n = 821* cells, respectively, from 3 animals). Bars represent mean ± SEM, data points represent individual animals

We next examined whether Shh signaling in differentiated astrocytes is restricted to those within the Gli1 lineage. We crossed *Gli1*^*CreER/+;*^*Ai14* mice with *Gli1*^*nlacZ/+*^ mice (*Gli1*^*CreER/nlacZ*^*;Ai14*) to generate mice in which we could distinguish between temporally distinct populations of cells expressing *Gli1*. We reasoned that because tamoxifen administered at P0 will indelibly mark progenitor cells expressing *Gli1* and their progeny, whereas βGal-expressing cells would reflect *Gli1* activity at the conclusion of the experiment, this approach would enable us to identify individual cells showing differential *Gli1* activity at two different time points within a single mouse. While this effectively produces a *Gli1* null mouse, *Gli1* is not required for Shh signaling during development and *Gli1* null mice show no developmental or behavioral deficits [21]. We administered tamoxifen to *Gli1*^*CreER/nlacZ*^*;Ai14* mice at P0 and analyzed tissues at P60 for colocalization of the tdTom and βGal reporter proteins. Although cells marked at P0 are found throughout all cortical layers, this analysis was restricted to deeper layers where βGal-expressing cells are mostly found. At P60, a large proportion (67%) of tdTom-labeled cells did not co-express βGal (Fig. 5), suggesting that in a substantial fraction of astrocytes in the Gli1 lineage, Shh signaling is downregulated sometime after P0. The remaining fraction of tdTom-labeled cells were double labeled with βGal (Fig. 5), suggesting either persistent Shh activity since birth or recurrence of Shh signaling in the same cell. Interestingly, 40% of βGal-labeled cells did not co-express tdTom (Fig. 5), suggesting Shh activity in differentiated astrocytes is not restricted to cells within the Gli1 lineage. Consistent with this, a greater number of cells express *Ptc* than *Gli1* in the adult cortex [9], indicating that *Gli1* activity reflects a fraction of cells capable of transducing Shh signal. Taken together, these data suggest that Shh signaling in differentiated astrocytes reflects recurrence of Shh activity in mature cells that is independent of developmental lineage.

## Discussion

Using genetic inducible fate mapping, we identified Shh signaling in a subpopulation of neonatal progenitor cells that generate cortical astrocytes. Shh activity in astrocyte progenitors is transient and is lost as astrocyte development proceeds. Astrocytes within the Gli1 lineage comprise half of all cortical astrocytes and are distributed broadly across superficial and deep cortical layers, demonstrating their substantial contribution to the cellular assembly of the cortex. Finally, our data show that while Shh signaling in astrocyte progenitor cells is transient, activity of the pathway recurs in a subpopulation of post-mitotic, differentiated astrocytes in a manner that is independent of cell lineage. Taken together, these data demonstrate a novel role for Shh signaling as a major contributor to astrocyte production during postnatal development, and further demonstrate its functional pleiotropy in cells along the astrocyte lineage.

One limitation of our study is the requirement for tamoxifen to trigger Cre-mediated recombination. It is possible that the dose and timing of tamoxifen may be insufficient to label all progenitor cells expressing *Gli1*. However in *Gli1*^*nlacZ/+*^ mice in which expression of the reporter protein is directly regulated by *Gli1* activity, we found that 30% of astrocyte progenitor cells express *Gli1*, consistent with the idea that Shh signaling is restricted to a subpopulation of astrocyte progenitors that is responsible for a defined lineage of cortical astrocytes. Alternatively, it is possible that this may instead reflect cells in different stages of development and competence for Shh signaling. A single i.p. injection of tamoxifen reaches its maximum concentration in the brain at 8 h post injection and is reduced by half by ~ 10 hours [28]. Because our experiments labeled cells with a single dose of tamoxifen at P0, activation of Shh signaling at later stages would fail to capture these cells. A constitutive *Cre* in the *Gli1* locus, rather than a tamoxifen inducible *Cre* as used in this study, could be informative for addressing this question. However the loss of temporal control over recombination would preclude specific analysis of neo- and perinatal glial progenitor cells since Shh signaling is active in the embryonic dorsal forebrain [29, 30]. Instead, future work using repeated tamoxifen dosing would provide further insight into this possibility. If indeed all astrocyte progenitors experience Shh signaling in a developmentally regulated manner, this would suggest that molecular and temporal cues may cooperate to define distinct pools of astrocyte progenitors.

Interestingly, we observed relatively fewer βGal-labeled cells in *Gli1*^*nlacZ/+*^ mice when compared to recombination-induced expression of the tdTom reporter. This suggests that the fraction of astrocyte progenitor cells expressing *Gli1* may be higher than our estimated 30%. While both *lacZ* and *CreER* expression are regulated by transcriptional activity of *Gli1*, the Ai14 reporter allele is highly recombinogenic and sensitive to even small amounts of Cre protein, whereas similar levels of *lacZ* expression may require higher amounts of βGal protein for antibody detection. Indeed, in our previous study, we found that antibody detection of βGal following Cre-induced *lacZ* expression was considerably weak at 1 week following recombination and required over 1 month to achieve full and robust antibody detection [9]. A more direct measure of *Gli1* activity, either by antibody staining or in situ hybridization approaches, would enable a more precise estimation of Shh activity in astrocyte progenitor cells. Unfortunately, antibody staining was not reliable in our hands (data not shown). Nevertheless, enzymatic detection of *lacZ* activity with X-Gal labeling is in agreement with our observation that only a fraction of cells in the dorsal SVZ express *Gli1* at P0 [23].

Cortical astrocytes have long been known to derive from radial glial and SVZ progenitor cells [13–15]. While many cells expressing βGal in *Gli1*^*nlacZ/+*^ tissues were observed in the dlSVZ, fate mapping studies suggest that radial glial progenitors may also express *Gli1*. At P3, marked cells with transitional morphologies, a single radial process extending towards the pial surface, are abundant throughout the cortex. Moreover, these cells co-label with vimentin, a widely used marker of radial glial cells [31, 32]. This suggests that Gli1 lineage astrocytes are derived from both dlSVZ and dorsal radial glial progenitors. In agreement with this, viral-mediated targeting of radial glia in neonatal brains demonstrates a requirement for Shh signaling in these cells for producing corpus callosum oligodendrocytes [18]. Taken together, these observations raise several interesting possibilities to consider. First, this suggests that Shh signals to two pools of progenitor cells that contribute astrocytes to the cortex. Whether these pools reflect distinct progenitor populations or cells along a developmental continuum is uncertain. Mapping the precise origins of anatomically or molecularly defined astrocyte populations and defining the relative contributions of each progenitor pool will shed new light on astrocyte diversification. Second, the observation reported by Tong et al [18] that Shh signals to radial glial progenitor cells responsible for contributing callosal oligodendrocytes suggests that astrocytes and oligodendrocytes may be derived from a common bipotent cell residing in the neonatal dorsal forebrain. Alternatively, Shh signals to two distinct progenitor populations, each committed to generating a specific class of glia. Distinguishing between these possibilities and dissecting the precise role of Shh signaling in each of these cell populations will be important for gaining a more complete understanding of glial development and cortical assembly. Interestingly, although loss of Shh signaling in embryonic and neonatal progenitors impairs oligodendrocyte production [18, 33], the number of astrocytes remains unchanged [3, 9]. This suggests that the Shh-responsive oligodendrocyte and astrocyte progenitors correspond to distinct populations, each with cell-type specific interpretations of Shh signal.

The precise role of Shh signaling in astrocyte development remains uncertain. However previous studies suggest that it may play a role in morphology. Selective deletion of *Smo* in Gfap-expressing cells from birth produces cellular hypertrophy and upregulation of GFAP expression in cortical astrocytes, in a manner consistent with mild reactive gliosis [9]. Cells also undergo dramatic changes in morphology, showing an increase in total branch length and number of higher order branches [3]. Interestingly, these conditional mutants show no difference in the total number of cortical astrocytes [3, 9]. One possibility is that progenitor cells from the non-Gli1 lineage compensate for any deficits in astrocytes from the Gli1 lineage. Indeed, conditional deletion of Shh signaling in embryonic dorsal oligodendrocyte progenitor cells (OPCs) depletes cortical oligodendrocytes in the embryo [29]. However these cells are eventually recovered from ventral OPC pools, demonstrating considerable cooperation between different progenitor populations to ensure the appropriate balance of cells. Similarly, dorsally-derived OPCs expand and ultimately compensate for loss of cells following ablation of ventrally-derived OPCs [34]. Although developing astrocytes exhibit more limited migration than OPCs and are deposited predominantly within the territories overlying their germinal zone [35], our identification of a mixed population of astrocyte progenitor cells within the dlSVZ suggests that the proper balance of astrocytes in the cortex could be achieved by cooperation between local progenitor pools.

The diversity of astrocytes has gained increasing recognition, but how such diversity is achieved is not well understood. One mechanism by which the CNS accomplishes cellular diversity is through the production of cells from distinct progenitor cell lineages. In the embryonic spinal cord and forebrain, astrocyte progenitor cells residing in molecularly and anatomically distinct domains produce regionally specified astrocytes that occupy the overlying territory defined by radial glial trajectories [35, 36]. Here, we show that Shh signaling is active in a subpopulation of astrocyte progenitor cells residing within the neonatal dlSVZ, suggesting that additional diversity exists among postnatal progenitor cells residing within a single domain. In the adult brain, Shh signaling defines molecular and physiological characteristics of mature astrocytes [37]. Our observations here suggest that Shh signaling may act earlier to diversify astrocytes and suggests that astrocyte heterogeneity emerges from the combinatorial actions of developmental lineage and local signaling.

Although fate mapping data show that cells within the Gli1 lineage are distributed throughout the cortex, *Gli1* activity in the cortex itself is considerably lower during the first few days after birth, suggesting that as Gli1 progenitor cells migrate away from the SVZ and into the cortex, they lose sensitivity to Shh signaling. This may reflect the availability of SHH to astrocyte progenitors. There are several potential sources of SHH in the neonatal and postnatal brain which include ventral forebrain neurons [20], cortical neurons [27, 38], epithelial cells [39], and the CSF [30, 40]. Because high levels of SHH are required to stimulate *Gli1* expression [21], our data suggest that *Gli1*-expressing progenitors are migrating away from their neonatal source of SHH, consistent with ventral forebrain neurons as the source of SHH to dlSVZ progenitors. Interestingly, although initially low, *Gli1* activity in the cortex increases progressively during the first two postnatal weeks. This is consistent with previous studies showing that Shh expression in the postnatal cortex is low at birth but increases until its peak at P14 [26]. Taken together, these data suggest that Shh signaling is highest in astrocyte progenitor cells at birth, and is subsequently lost in immature astrocytes residing in the cortex. However a subpopulation of mature, differentiated astrocytes experience a recurrence of Shh signaling from local cortical neuronal sources. Interestingly, the pattern of Shh activity in mature astrocytes is distinct from the distribution of cells in the Gli1 lineage, suggesting that pathway activity in progenitor cells and mature astrocytes occurs independently. Taken together, these data suggest that Shh signaling segregates astrocyte populations by both lineage and local activity.

Shh signaling regulates a diverse repertoire of cellular activities in multiple cell types, including neural stem and progenitor cells, neurons, and astrocytes [41]. It is likely that the molecular programs initiated by Shh activity in astrocyte progenitors and mature astrocytes confer distinct functional characteristics that are cell-type dependent. This application of a single molecular pathway in cell type dependent ways reflects the pleiotropic nature of Shh signaling and demonstrates the remarkable capacity of the pathway to be deployed in a broad array of cellular activities.

## Conclusions

In this study, we demonstrate that Shh signaling identifies a subpopulation of neonatal glial progenitor cells responsible for generating cortical astrocytes. Shh signaling declines as cells undergo maturation, but recurs in a discrete subpopulation of mature astrocytes, independent of lineage. These data establish Shh signaling as a major contributor to postnatal astrocyte development and further, demonstrates that distinct populations of cortical astrocytes are defined by Shh signaling.

## Materials and methods

### Animals

The following transgenic mouse lines were used: *Gli1*^*nLacZ/+*,21^, *Gli1*^*CreER/+*,19^, and *R26*^*tdTom/tdTom*^ (Ai14) [42]. Animals were maintained on a 12 h light/dark cycle and given access to food and water ad libitum. All experiments were conducted in accordance with the Drexel University Institute for Animal Care and Use Committee. Male and female animals between postnatal day (P)0 and P60 were used. No differences were observed so data were pooled.

### Tamoxifen

Tamoxifen (Sigma, T5648-1G) was diluted to a final concentration of 5 mg/ml or 10 mg/mL in corn oil. P0 *Gli1*^*CreER/+*^*;Ai14* mice received 50 mg/kg tamoxifen by intragastric injection for 1 or 3 days consecutively and tissue was harvested at various indicated time points. All other *Gli1*^*CreER/+*^*;Ai14* mice received 100 mg/kg of tamoxifen by intragastric injection (P3), subcutaneous injection (P7), or oral gavage (P14 and above) for 1 or 3 days consecutively, and tissue was harvested at indicated time points. For adult comparisons, *Gli1*^*CreER/+*^*;Ai14* mice greater than 2 months old received 250 mg/kg of tamoxifen by oral gavage for 1 or 3 days consecutively, and tissue was harvested 2 weeks later, unless otherwise noted.

*BrdU.* BrdU (Sigma, B9285-1G) was dissolved in 0.007 N NaOH in sterile saline and administered via intraperitoneal (i.p.) injection. For long term experiments, mice received 50 mg/kg in mice ages P0 to P14 or 200 mg/kg at P28 and older, at 6–24 h after tamoxifen.

### Perfusion and histology

Animals were given an i.p. injection of a Ketamine/Xylazene/Acepromazine cocktail and transcardially perfused with 10 mM PBS followed by 4% paraformaldehyde solution. Brains were dissected and post-fixed in 4% paraformaldehyde for 4–6 h followed by cryoprotection in 30% sucrose and stored at 4 °C for at least 48 h or until ready for sectioning. Brains were sectioned on a cryostat (Leica CM3050S, Wetzlar, Germany) at 40 μm and serial sections were collected in a 96 well plate and stored at 4 °C in TBS with 0.05% sodium azide. Sections were processed by free floating immunohistochemistry. For P0 and P3 samples, brains were embedded in OCT medium after cryoprotection and stored at − 20 °C until sectioning. Tissues were sectioned by cryostat at 16–20 μm and collected directly onto coated slides and stored at − 80 °C, protected from air and light. Immunohistochemistry was performed using the following primary antibodies for fluorescence: rabbit anti-βgal (1:1 k/1:10kMP Biomedicals), chicken anti-βgal (1:1 k, Abcam), mouse anti-BLBP (1:1 k, Abcam), sheep anti-BrdU (1:500, Maine Biotechnology Services), mouse anti-CC1 (1:1 k, Calbiochem), rabbit anti-RFP (1:500, MBLI), rabbit anti-S100β (1:1 k, DAKO), goat anti-Sox9 (1:1000, R&D), and chicken anti-Vimentin (1:1 k, Invitrogen). For BrdU staining, tissue was pre-incubated in 2 N HCl for 30 min and neutralized with 0.1 M TBS before incubation in block and primary antibody. Fluorescent labeling was achieved using species-specific AlexaFluor-tagged secondary antibodies, Alexa Fluor 488, Alexa Fluor 568, or Alexa Fluor 647 (1:1 k, Life Technologies), followed by counterstaining with DAPI (1:36 k, Life Technologies). For brightfield immunostaining, tissues were quenched in TBS with 0.3% H_2_O_2_ and 30% methanol for 30 min prior to incubation in block and primary antibody. The following antibodies were used: rabbit anti-βgal (1:40 K, MP Biomedicals) and rabbit anti-RFP (1:500, MBLI). For brightfield staining, species-specific biotinylated secondary antibodies (Vector) were used at 1:400 followed by incubation in avidin-biotin complex (ABC, Vector). Visualization was achieved using 3′-3 diaminobezedine (DAB, Vector) as the developing agent. 4% paraformaldehyde post-fix was applied to all slide-mounted tissue for 30 min prior to staining.

### Microscopy

Stained sections were examined and imaged in brightfield and fluorescence using an upright microscope (Zeiss) and StereoInvestigator software (MBF Biosciences). Confocal images were obtained on an inverted microscope (Leica) using LAS X software at 20x, 40x oil or 63x oil objectives, with a 1 μm slice distance.

### Cell quantification

Stereological estimates of the total number of cells were obtained in tissues stained for brightfield microscopy. The cortex was analyzed in a series of 6–8 sections, sampled every 480 μm, and bounded by the midline dorsally and the end of the external capsule ventrally. Anterior and posterior boundaries were defined as Fig. 22 and Fig. 44, respectively from Paxinos and Franklin [43]. We used a modified optical fractionator and stereological image analysis software (StereoInvestigator, MBF Bioscience) operating a computer-driven stage attached to an upright microscope (Zeiss). The cortical area to be analyzed was outlined at low magnification, and counting frames were selected at random by the image analysis software. Cells were counted using a 40x objective and DIC optics. A target cell count of 300 cells was used to define scan grid and counting frame sizes, with a 2 μm guard zone. For all analyses, only cells with a clear and distinct labeled cell body were analyzed. Analysis of βgal cells in the cortex and dlSVZ of P0 tissues was performed on slide mounted tissues stained for brightfield microscopy. Each region was traced in Neurolucida (MBF Bioscience) at 5x and individual βgal-labeled cells were mapped across 1–2 sections with separate markers in each region at 40x using DIC optics. Single-cell analysis of co-labeling was evaluated on double-stained immunofluorescent tissues by taking confocal sections with a 1um slice distance. For each cortex sampled, multiple z stacks were collected from 3 to 5 sections within the anterior and posterior boundaries defined by Fig. 22 and Fig. 44, respectively from Paxinos and Franklin (2013). A minimum of 150 cells per animal were analyzed and data were averaged per animal.

### Statistics

All statistical analyses were performed using Prism 8 (GraphPad). We performed unpaired Student’s t-tests or one-way ANOVA, as appropriate. Specific analyses for each dataset are indicated in corresponding figure legends. For single cell analyses, data were averaged per animal and statistical tests were performed by analysis of individual animals.

## Supplementary Information

**Additional file 1 Supplemental Fig. 1. Marked cells show characteristics of transitional radial glia. (A)** Brightfield immunostaining for RFP in the cortex of a mouse at P3 after receiving tamoxifen at P0 showing many cells with transitional morphologies and the appearance of residual radial glial fibers. Scale bar, 25 μm **(B-F)** Colocalization of tdTom (C, red), vimentin (D, green), and BrdU (E, gray) in the cortex of *Gli1*^*CreER/+*^;Ai14 mice at P3 after tamoxifen at P0. Merged image in (B), single channel images in C-F.

## Data Availability

All data and reagents are available upon request.
