# Supplementary material for: A subpopulation of astrocyte progenitors defined by Sonic hedgehog signaling
Source: Neural Dev. 2022 Jan 14;17:2. doi: 10.1186/s13064-021-00158-w (PMC8759290; doi:10.1186/s13064-021-00158-w)
Supplement: Supplementary file 1 — Additional file 1 Supplemental Fig. 1. Marked cells show characteristics of transitional radial glia. (A) Brightfield immunostaining for RFP in the cortex of a mouse at P3 after receiving tamoxifen at P0 showing many cells with transitional morphologies and the appearance of residual radial glial fibers. Scale bar, 25 μm (B-F) Colocalization of tdTom (C, red), vimentin (D, green), and BrdU (E, gray) in the cortex of Gli1CreER/+;Ai14 mice at P3 after tamoxifen at P0. Merged image in (B), single channel images in C-F. [file 13064_2021_158_MOESM1_ESM.pdf]

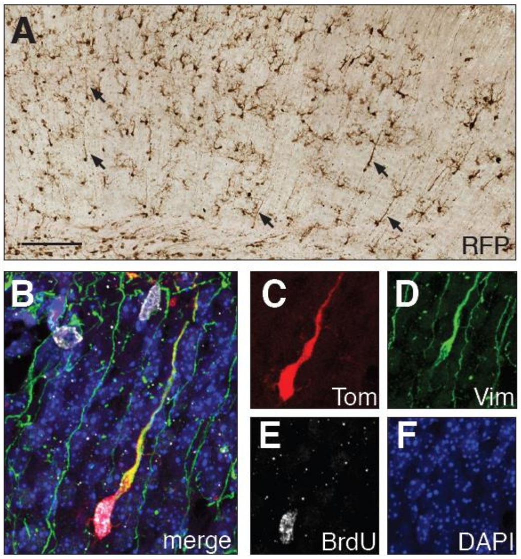

Supplemental Figure 1. Marked cells show characteristics of transitional radial glia. (A) Brightfield immunostaining for RFP in the cortex of a mouse at P3 after receiving tamoxifen at P0 showing many cells with transitional morphologies and the appearance of residual radial glial fibers. Scale bar, 25 µm **(B-F)** Colocalization of tdTom (C, red), vimentin (D, green), and BrdU (E, gray) in the cortex of *Gli1*$^{CreER/+}$;Ai14 mice at P3 after tamoxifen at P0. Merged image in (B), single channel images in C-F.
